# The co-occurrence of water insecurity and food insecurity among Daasanach pastoralists in northern Kenya

**DOI:** 10.1017/S1368980022001689

**Published:** 2023-03

**Authors:** Hilary J Bethancourt, Zane S Swanson, Rosemary Nzunza, Sera L Young, Luke Lomeiku, Matthew J Douglass, David R Braun, Emmanuel K Ndiema, Herman Pontzer, Asher Yoel Rosinger

**Affiliations:** 1 Department of Anthropology, Northwestern University, Evanston, IL, USA; 2 Institute for Research Policy, Northwestern University, Evanston, IL, USA; 3 Department of Evolutionary Anthropology, Duke University, Durham, NC, USA; 4 Kenya Medical Research Institute (KEMRI), Nairobi, Kenya; 5 Department of Education and Outreach Programs, National Museums of Kenya, Nairobi, Kenya; 6 College of Agricultural Sciences and Natural Resources and Agricultural Research Division, University of Nebraska-Lincoln, Lincoln, NE, USA; 7 Center for the Advanced Study of Human Paleobiology, Department of Anthropology, The George Washington University, Washington, DC, USA; 8 Department of Human Evolution, Max Planck Institute of Evolutionary Anthropology, Leipzig, Germany; 9 Department of Earth Sciences, National Museums of Kenya, Nairobi, Kenya; 10 Global Health Institute, Duke University, Durham, NC, USA; 11 Department of Biobehavioral Health, The Pennsylvania State University, 219 Biobehavioral Health Building, University Park, PA 16802, USA; 12 Department of Anthropology, The Pennsylvania State University, University Park, PA, USA

**Keywords:** Water scarcity, Water insecurity, Food insecurity, Socio-economic status, Marginalised populations, Climate change, Pastoralists

## Abstract

**Objective:**

Water plays a critical role in the production of food and preparation of nutritious meals, yet few studies have examined the relationship between water and food insecurity. The primary objective of this study, therefore, was to examine how experiences of household water insecurity (HWI) relate to experiences of household food insecurity (HFI) among a pastoralist population living in an arid, water-stressed region of northern Kenya.

**Design:**

We implemented the twelve-item Household Water Insecurity Experiences (HWISE, range 0–36) Scale and the nine-item Household Food Insecurity Access Scale (HFIAS, range 0–27) in a cross-sectional survey to measure HWI and HFI, respectively. Data on socio-demographic characteristics and intake of meat and dairy in the prior week were collected as covariates of interest.

**Setting:**

Northern Kenya, June–July 2019.

**Participants:**

Daasanach pastoralist households (*n* 136) from seven communities.

**Results:**

In the prior 4 weeks, 93·4 % and 98·5 % of households had experienced moderate-to-severe HWI and HFI, respectively. Multiple linear regression analyses indicated a strong association between HWI and HFI. Each point higher HWISE score was associated with a 0·44-point (95 % CI: 0·22, 0·66, *P* = 0·003) higher HFIAS score adjusting for socio-economic status and other covariates.

**Conclusions:**

These findings demonstrate high prevalence and co-occurrence of HWI and HFI among Daasanach pastoralists in northern Kenya. This study highlights the need to address HWI and HFI simultaneously when developing policies and interventions to improve the nutritional well-being of populations whose subsistence is closely tied to water availability and access.

Approximately 2·37 billion people around the globe suffered from moderate-to-severe food insecurity (FI) in 2020, meaning they lacked reliable access to sufficient quantities and quality (i.e. nutritious) of food^(1)^. Water insecurity (WI), the lack of reliable access to safe, sufficient water for everyday needs^(2)^, may be even more prevalent. Approximately two billion people worldwide lacked access to safely managed drinking water in 2020^(3)^, and approximately four billion face severe water scarcity at least 1 month out of the year^(4)^. The syndemic – or co-occurring and mutually amplifying – relationship between these two forms of insecurities has received growing attention^(5–9)^. Yet limited research has examined the ways in which co-occurrence of these two insecurities manifests across different populations^(9–12)^. Given the vital role water plays in the production of food and preparation of nutritious meals, it is valuable to understand how problems with access to sufficient water may be a contributing factor to insufficient food quantity and quality.

A handful of qualitative studies have highlighted some of the pathways through which WI may contribute to reduced food quantity and/or quality in low- and middle-income settings. For example, water scarcity may limit the ability to grow and tend to a garden^(8,13–15)^ or create challenges for raising livestock^(13)^. Insufficient amounts of water may hinder the ability to cook available foods or force a shift from more nutritious but water-intense foods (e.g. beans and whole grains) to those requiring less water (e.g. tubers or milled grain porridge)^(8,10,13,16,17)^. Furthermore, for economically disadvantaged households, the need to purchase water may result in less money available for food purchases^(15,16,18)^.

WI may also impact experiences of FI through less direct pathways. For instance, hunger may increase if the physical exertion of collecting and carrying water substantially increases energy expenditure^(19)^. Time engaged in water procurement may also compromise income-generating opportunities that could fund food purchases^(13,16–18)^.

While a number of studies have demonstrated quantitatively the positive relationships between FI and measures of WI^(9–12,20)^, time spent collecting water^(21)^ and water scarcity^(22)^, we are still far from having a comprehensive understanding of the degree to which WI and FI co-occur in different contexts. For example, two studies combining data from different study sites in low-and middle-income countries around the world noted a strong relationship between household experiences of WI and household experiences of FI^(10,11)^. However, it was clear that the magnitude of the relationship varied across study sites^(10)^, suggesting that other contextual factors, such as subsistence patterns, climate and water infrastructure, may be at play in shaping the degree to which WI exacerbates FI. Similarly, in a longitudinal study of postpartum women in Kenya, FI was related to preceding WI in some but not all months, suggesting that the relationship may differ by season. Drought and climate variability might also influence the magnitude of association between WI and FI and the types of coping strategies employed^(22)^. Moreover, most of the existing studies report only bivariate correlations between WI and FI^(9,12,20–22)^. Only a few known studies adjusted for measures of financial and social capital, such as wealth^(23)^, social status^(10)^ and water expenditures^(11)^. Measures of socio-economic status (SES) are inversely associated with WI^(11,18,22,24,25)^ and FI^(10,23,25)^, but it remains unclear to what degree SES confounds relationships between WI and FI.

Self-subsistent populations, especially those living in arid environments with little to no water infrastructure, may be particularly vulnerable to experiencing concurrent WI and FI. Pastoralist populations, for example, have a particularly high prevalence of FI^(26,27)^, as they tend to have fewer assets and income-generating opportunities and lower access to basic services, including water services and social safety nets^(28,29)^. Water plays a crucial role in the ability to maintain large livestock herds that provide a direct source of protein-rich food (e.g. milk) and are a primary source of income or trading opportunities for acquiring other food staples. Livestock also continue to be a valuable resource to exchange in social networks of bond partnership^(30)^. Yet livestock may also be a liability with regard to WI^(31)^, as substantial amounts of water are required to keep livestock alive and healthy enough to sell or trade for a decent value or use as a source of food^(13)^. Water scarcity, drought and seasonal fluctuations in rainfall are recognised as major contributors to FI among pastoralists in eastern Africa^(32–34)^. However, the co-occurrence of WI and FI among pastoralists has not, to our knowledge, been quantified. This quantification is important for recognising the overlooked needs of such underserved populations.

The objective of this study, therefore, was to examine the relationship between household WI (HWI) and household FI (HFI) among Daasanach pastoralists in northern Kenya. We approached this objective in three ways. First, we tested how HWI and HFI relate to each other using experience-based scales; we hypothesised that more frequent experiences of HWI would be associated with more frequent experiences of HFI. Second, we tested how HFI related to the experience of having to change what was eaten due to water problems independently of other HWI experiences. Third, we examined how HWI related to the frequency of consuming milk, traditionally an important source of sustenance and protein for pastoralists^(35)^.

## Methods

### Study population

Data for this study come from the Daasanach Human Biology Project. Daasanach are semi-nomadic pastoralists who inhabit a region that surrounds the northeastern shore of Lake Turkana in northern Kenya and the Omo River Valley region of southern Ethiopia^(36)^. In June and July 2019, we surveyed households from seven Kenyan Daasanach communities. Six of the communities were relatively permanent settlements at varying distances from the town of Illeret, which is located approximately 3·5 km from the eastern Lake Turkana shore. One of the seven communities was nomadic, travelling with their livestock farther southeast of the lake.

The region inhabited by Daasanach in northern Kenya is arid, with bimodal patterns of rainfall (ranging from 120 to 500 mm/year and averaging around 130–217 mm/year^(37,38)^) and frequent droughts^(31)^, making agricultural production difficult. The maize, beans and sorghum that provide the main source of calories for Daasanach living closer to Illeret generally must be purchased or obtained through trade from small stores or passing merchants. Milk and occasionally meat from goats, sheep, cattle and, occasionally, camels supplement the diets of those living near Illeret. For nomadic households, milk may be a primary food source when they remain away from areas of commerce for long stretches of time.

Even in the semi-permanent communities sampled, access to piped water, sanitation and electricity was extremely limited for most Daasanach households. The primary source of drinking water used by households was hand-dug wells varying from ∼0·3 to 1·5 m in depth situated in the dry riverbeds that drain into Lake Turkana. Although several standpipes in Illeret piped water from a shallow flood plain beside Lake Turkana, the standpipe water was described by residents as unpleasantly salty and was only consumed in small quantities if well water was unavailable^(39)^. Moreover, unlike other sources, it costs money to collect standpipe water. A borewell and pond were used by the nomadic community in our sample, but the distance to either water source from their temporary residence was > 2 km away. Travelling to collect water at these remote sites also posed a safety risk, as lethal conflicts with neighbouring tribes over water, livestock and pasture lands were reportedly common in remote regions where territorial boundaries were blurred.

Thus, accessing water was a laborious activity for most households. Multiple hours were spent each day making 2–3 trips per day to a water source. For each trip, it was necessary to wait one’s turn to collect water, sometimes having to re-dig or clean out the well and wait for water to resurface. The amount of water that could be collected at one time was often limited to what could be carried by the women or children responsible for the task; only a few households (primarily in nomadic communities) had donkeys to help haul water. Limited number of water-holding vessels also meant only small amounts of water could be stored at any given time.

### Participant recruitment

Prior to data collection, permission was obtained from the Director of Health in the county government of Marsabit, Kenya and community leaders in all Daasanach communities sampled. We worked with local Daasanach translators to translate the survey instruments into Daasanach. Translation was conducted by one translator and reviewed by two other translators. Any disagreements on translations were discussed until a final translation was agreed upon.

We worked with local elders, community health assistants and community health volunteers to recruit participating households from the seven communities. Every third house in the community was selected to minimise familial clusters. The number of households sampled in each community was based on feasibility, that is, the number of families we could survey in the pre-specified number of days allocated for each location. These time restrictions resulted in sampling between 12 and 28 households per community for a total of 136 households across the seven communities. All participants provided verbal informed consent.

For each household, we invited both the male and female household heads (if present) to participate in the surveys on HWI, HFI and other household characteristics. All survey components were administered by the study team with the help of Daasanach translators.

In 77·2 % (*n* 105) of the households, both household heads responded to the surveys; female household heads were the primary respondents in 21·3 % (*n* 29) of households, and male household heads were the primary respondents in 1·5 % (*n* 2) of households. Survey responses did not differ depending on whether both household heads or only the female household head responded to the survey (i.e. scores on the instruments described below were similar, data not shown).

### Primary outcome variable of interest: household food insecurity (HFI) experiences

HFI was measured using the nine-item Household Food Insecurity Access Scale (HFIAS)^(40)^. This scale addresses the frequency in the previous 4 weeks with which any household member experienced anxiety and uncertainty of food access, inadequacy of food quality, insufficient food intake and hunger^(40)^. A score of 0, 1, 2 or 3 was given to answers of never, rarely (1–2 times), sometimes (3–10 times) or often/always (11+ times), respectively, for a score range of 0–27. Internal consistency (Cronbach’s alpha) for the nine HFIAS items in our sample was 0·896. We assessed the number of households considered to have experienced moderate or severe HFI based on standard scoring procedures^(40)^ of affirmative responses to more extreme food access questions (e.g. having to skip meals or go an entire day or night without food; see Supplemental Text 1 for survey questions and scoring details).

### Secondary outcome variable of interest: frequency of milk intake

We measured the frequency of milk intake by asking each adult respondent how frequently they had consumed milk in the previous week: zero times per week, several times per week, once per day or multiple times per day. We used the maximum frequency reported for either household head and created a categorical variable indicating either no consumption of milk, less than daily consumption of milk or daily consumption of milk.

### Primary predictor variables

#### Household water insecurity (HWI) experiences

HWI was measured using the twelve-item Household Water Insecurity Experience (HWISE) Scale validated in multiple languages among other low-income populations for quantifying experiences of water access, use and stability^(41)^. The twelve questions ask households about the frequency in the prior 4 weeks that any household member experienced the following phenomena: (a) emotional distress (worry, anger or shame) related to their water situation; (b) disruptions in daily life due to water problems (had to change what was eaten; were unable to wash clothes, body or hands; had to change plans for the day); (c) physiological discomfort due to not having enough water (unable to drink as much water as desired or going to bed thirsty) or (d) interruptions in the water supply or no usable water in the household whatsoever (see Supplemental Text 2 for complete English phrasing of survey questions and details on scoring the HWISE scale). Cronbach’s alpha for the twelve HWISE items in our sample was 0·878. The questions used the same response options and scores as the HFIAS scale, with a score range of 0–36. Although a score ≥ 12 has been used as a provisional cut-off for defining water insecurity^(41)^, only nine households had scores < 12. Thus, for descriptive purposes, we calculated the proportion of households with HWISE scores between 12 and 23 or ≥ 24.

To understand how water-induced changes in food choice related to HFI, we assessed responses to the single HWISE item on food, which asks, ‘In the last 4 weeks, how frequently have you or anyone in your household had to change what was being eaten because there were problems with water (e.g. for washing foods, cooking, etc.)?’ As only twelve households answered ‘never’ and only 18 households answered ‘often/always,’ we dichotomised this variable, grouping responses of ‘never’ with ‘rarely’ and ‘sometimes’ with ‘often/always’. To control for water-related disruptions in life that were not directly related to food, we then created an HWISE-11 score, which was the sum of scores for the remaining eleven items after excluding the food item.

### Covariates

We adjusted for the age of the female household head, as women and girls are responsible for collecting water in most Daasanach households, and the job of water collection may be more burdensome at older ages. In two households in which the male household head was the only adult interviewed, we used the age of the male household head as a substitute.

We also adjusted for the number of children (< 16 years) in the household, which is likely to affect both HWI and HFI^(11,42)^.

As income is often obtained only sporadically among Daasanach, it is not reflective of one’s earning potential, wealth, resources and social capital. All of these may influence both HWI and HFI and may mediate the relationship between the two. Both financial and social capital have, for example, been associated with lower WI in other Kenyan pastoralist populations^(31)^. Therefore, to obtain a summary index of SES, we performed a principal component analysis (PCA) with variables for reported income (Kenyan shillings and natural log-transformed) earned in the last month; livestock wealth (the summed market value of all goats, sheep, cattle, camels and chickens owned by the household, natural log-transformed) and self-perceived social status. The latter was measured by asking household heads to rank themselves on a MacArthur ladder^(43)^ from 1 (worst off) to 10 (best off) relative to others in their community regarding income, education and social status. For households with two household heads present for the interview, we used the average of each of their self-rankings. The first component of the PCA explained 56·2 % of the variation in the three variables. We used the score of that first PCA component as our SES measure in our statistical models. Two households were missing data on perceived social status. For those two households, we computed an SES score from a second PCA performed with natural log-transformed household income and livestock wealth only.

Mobility (e.g. ability to bring livestock to regions with more water and pasture or be closer to social support networks) may be a mode of resilience against HWI and HFI for Daasanach. Household heads were asked about the number of times they had moved in the previous year. This variable was right-skewed, with two households reported moving more than twenty times; we truncated this variable at 20.

In addition to HWI experiences, we asked participants about how much time was required for a single trip to collect water (including queue time) and how many water trips they took per day. Water fetch time was calculated by multiplying the number of minutes required per water collection trip times the number of trips made per day.

Although we collected GPS data on the distance between each household and the nearest water source, we did not include this measure as a covariate because it is strongly correlated with the community.

### Statistical analyses

All analyses were performed using Stata (v17·0).

For descriptive purposes, we examined summary statistics for HFIAS score, HWISE score and each of the covariates among the full sample and by the community.

To test the relationship between experiences of HWI and HFI, we used multivariable linear regression models with standard errors clustered on community that regressed (separately) scores for HFIAS (modeled continuously) on HWISE score (modeled continuously). We accounted for unobserved heterogeneity between communities by controlling for community fixed effects instead of random effects, as random effects variance can be unstable with fewer than eight groups^(44)^. We also clustered standard errors on community to account for any potential correlation of observations within communities. We adjusted for covariates in a stepwise fashion and tested model fit using likelihood ratio tests. The base model regressed HFIAS on HWISE score adjusting only for the community. We then added adjustment for the PCA-derived SES score. Finally, we added female household head age, number of children in the household, times moved in the previous year and daily water collection time. We tested for a quadratic relationship with age; since there was no evidence of age having a curvilinear relationship with HFI, we retained only the linear term. Using the fully adjusted models, we used Stata’s post-estimation margins command to estimate and graph-predicted scores for HFIAS across the range of HWISE scores^(45)^.

The same model was used to test how water-induced food choice changes independent of other problems experienced with water, but this time HFIAS score was regressed on the bivariate measure of having to sometimes/often (relative to never/rarely) change what was being eaten because of water problems and the summed score of the remaining eleven HWISE items, controlling for community and covariates.

Finally, to test the relationship between HWI and frequency of milk intake, we built an ordered logistic regression model to test for the association between HWISE score and the odds of drinking milk less than daily or daily relative to never in the previous week controlling for community fixed effects and all covariates. This analysis was restricted to the six semi-permanent communities, as all households in the nomadic community drank milk daily.

## Results

### Household characteristics in overall sample and within communities

Mean (sd) age of the female household head was 34·8 (12·5), and households had an average of 4·5 (2·5) resident children (Table 1). Across communities, mean HFIAS and HWISE scores were 17·3 (5·2) and 20·2 (6·8), respectively. Most households reported experiencing substantial HFI and HWI in the previous 4 weeks, with 98·5 % of households classified as moderately-to-severely food insecure and 93·4 % having HWISE scores ≥ 12. Two-thirds of the households reported having to change what was being eaten because of water problems ‘sometimes’ or ‘often.’ Only 39·0 % of households reported consuming milk daily in the previous week.

**Table 1:** Food insecurity, water insecurity and socio-demographic characteristics of Daasanach households participating in the Daasanach Health and Life History Project 2019 (*n* 136)

	% of households	Mean	sd ^*^	Interquartile range	*n* of households
Indicators of food insecurity					
HFIAS score (range: 0–27)		17·3	5·2	15, 21	
Severity of household food insecurity					
Mild^*^	1·5 %				2
Moderate^*^	2·2 %				3
Severe^*^	96·3 %				131
Frequency of milk intake in previous week					
None	38·2 %				52
Less than daily	22·8 %				31
Daily	39·0 %				53
Indicators of water insecurity					
HWISE score (range: 0–36)		20·2	6·8	16, 25	
HWISE score 12–23 pts	59·6 %				81
HWISE score 24–36 pts	33·8 %				46
Frequency in previous 4 weeks of having to change what was eaten because of water problems					
Never/rarely (0–2 times)	35·3 %				48
Sometimes/often/always (3+ times)	64·7 %				88
h/d spent fetching water		4·3	2·3	2, 6	
Socio-demographic characteristics					
Household income in previous month (median, USD)^†^	5			0, 26	
Household monetary value of livestock (median, USD)^†^	713			338, 1358	
Perceived social status (range: 1–10/worst-best)^‡^		3·3	2·2	2, 5	
Number of children < 16 years in household		4·5	2·5	3, 6	
Female household head age (years)		34·8	12·5	25, 42	
Number of times moved in the previous year (median)		4·1	6·1	0, 6	

HFIAS, Household Food Insecurity and Access Scale; HWISE, Household Water Insecurity Experience.

* Classification for mild, moderate and severe household food insecurity is based on affirmative responses to more severe HFIAS items. See Supplemental Text 1 for details on scoring and classification definitions.

† Medians instead of means reported for household income and livestock wealth due to their skewed distributions.

‡ Two households were missing data on self-perceived social status.

Household characteristics varied by the community (online Supplemental Table 1). The mean number of times households moved in the previous year increased with greater distance from the town; the nomadic community households reported moving monthly on average. Although the distance from the nearest water source ranged from a mean of 358 to 2766 m in the nomadic community, there was little variation in the mean number of hours per day spent fetching water. Community mean HFIAS scores ranged from 14·0 (7·3) to 20·8 (3·2), and 100 % of households were classified as severely FI in four communities. Mean HWISE scores ranged from 15·3 (8·4) to 24·8 (3·8). The percentage of households reporting having to change what was eaten due to water problems varied across communities from 33·3 % to 92·3 %. The percentage of households reporting daily milk consumption ranged from a low of 7·1 % in the community closest to the nearest commercial town to a high of 100 % in the nomadic community.

### Relationship between household water insecurity and household food insecurity experiences

**Fig. 1:**
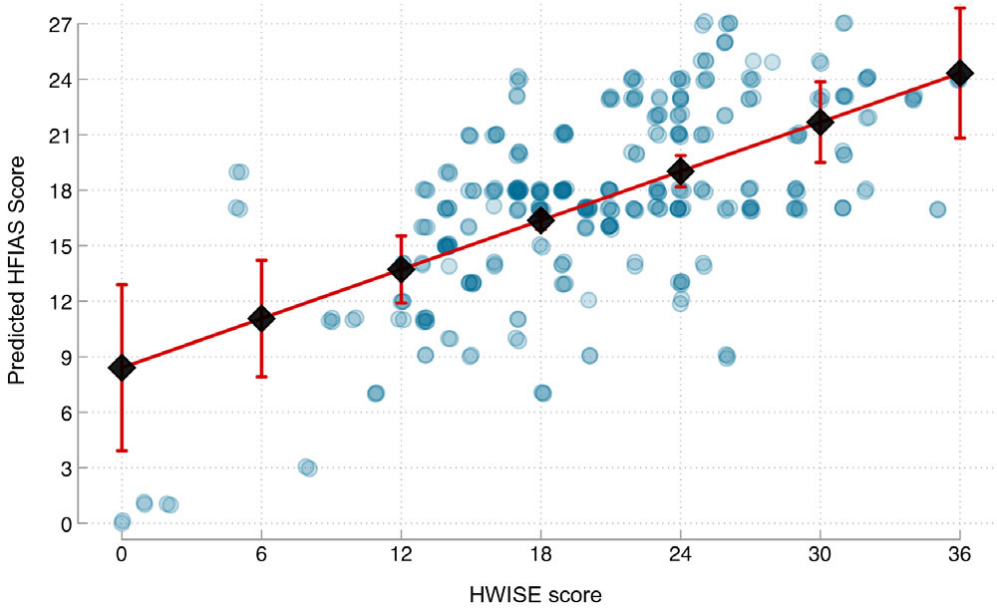
Predicted HFIAS scores in relation to HWISE scores. Note: HFIAS, Household Food Insecurity Access Scale; HWISE, Household Water Insecurity Experiences scale; SES, socio-economic status. Observed scores (blue circles) and predicted scores and 95 % CI (black lines with error bars) of HFIAS (range 0–27) in relation to HWISE score (range 0–36). Predicted values estimated from multivariable linear regression models with standard errors clustered on community and adjusting for community-fixed effects, PCA-derived SES score, age of female household head, number of children in household, number of times moved in the previous year and h/d spent collecting water

HWI experiences were strongly positively related to HFI experiences independently of community, SES and other covariates (Table 2; Fig. 1). Each point higher HWISE score was associated with an average 0·44-point (95 % CI: 0·22, 0·66, *P* = 0·0028) higher HFIAS score. Adding the PCA-derived SES score to the base model improved fit (*P* of likelihood ratio test = 0·0028), and SES score was inversely associated with HFIAS score. Model fit was not improved by adding the other covariates (female household head age, number of children in the household, times moved in the previous year, or water fetching time, *P* of likelihood ratio test = 0·72). The fully-adjusted model estimated that at the median HWISE score of 20, well above the threshold generally used to indicate water insecurity, households were predicted to have an HFIAS score of around 17·25 (95 % CI: 17·21, 17·29) (Fig. 1).

**Table 2:** Results from multivariable linear regression models testing HFIAS score in relation to HWISE score (*n* 136 households)

	*β*	95 % CI	*P*	Adj R^2^	*β*	95 % CI	*P*	Adj R^2^	*β*	95 % CI	*P*	Adj R^2^
HWISE score	0·47	0·24, 0·71	0·0025		0·47	0·27, 0·66	0·0011		0·44	0·22, 0·66	0·0028	
PCA-derived SES score*					−0·84	−1·61, –0·07	0·037		−0·81	−1·60, –0·02	0·047	
Age of female household head (years)									0·035	−0·033, 0·10	0·25	
Number of children in household									0·045	−0·35, 0·44	0·79	
Times moved in the previous years									0·049	−0·22, 0·32	0·67	
h/d spent collecting water									0·054	−0·29, 0·40	0·72	
				0·466				0·496				0·487

HFIAS, Household Food Insecurity and Access Scale; HWISE, Household Water Insecurity Experience; PCA, principal component analysis; SES, socio-economic status.

Results from multivariable linear regression models with SE clustered on community and adjusting for community fixed effects (community coefficients not shown). The baseline model adjusts only for the community. Model 2 adds the PCA-derived SES score. Model 3 adds the covariates for age of female household head, number of children in household, number of times moved in the previous year and h/d spent collecting water

* SES score was derived from the first component of a principal component analysis (PCA) conducted with ln(household monthly income+1), ln(livestock wealth+1) and average perceived social status score of household heads (ranging from 1 to 10).

### Household Food Insecurity experiences in relation to changing what was eaten due to water problems

Households reporting sometimes or often having to change what was eaten due to water problems in the previous 4 weeks had HFIAS scores that were on average 1·78 points (95 % CI: 0·41, 3·14 *P* = 0·019) higher adjusting for other aspects of HWI (Table 3).

**Table 3:** Results from multivariable linear regression models testing HFIAS score in relation to HWISE score separated into the item on food (sometimes/often *v*. never/rarely having to change what was eaten due to water problems) and the remaining eleven items (*n* 136 households)

	*β*	95 % CI	*P*	Adj R^2^	*β*	95 % CI	*P*	Adj R^2^	*β*	95 % CI	*P*	Adj R^2^
Sometimes/often changing what was eaten due to water problems (reference: never/rarely changing what was eaten)	2·14	0·55, 3·74	0·017		1·84	0·40, 3·28	0·021		1·78	0·41, 3·14	0·019	
11-item HWISE score*	0·44	0·19, 0·69	0·0049		0·44	0·23, 0·66	0·0021		0·42	0·18, 0·67	0·0055	
PCA-derived SES score^†^					−0·80	−1·58, –0·03	0·044		−0·76	−1·56, 0·04	0·059	
Age of female household head (years)									0·039	−0·027, 0·10	0·20	
Number of children in household									0·035	−0·39, 0·46	0·85	
Times moved in the previous years									0·047	−0·21, 0·30	0·66	
h/d spent collecting water									0·032	−0·33, 0·39	0·83	
				0·469				0·496				0·488

HFIAS, Household Food Insecurity and Access Scale; HWISE, Household Water Insecurity Experience; PCA, principal component analysis; SES, socio-economic status.

Results from multivariable linear regression models with SE clustered on community and adjusting for community fixed effects (community coefficients not shown). The baseline model adjusts only for the community. Model 2 adds the PCA-derived SES score. Model 3 adds the covariates for age of female household head, number of children in household, number of times moved in the previous year and h/d spent collecting water.

* Eleven-item HWISE score is the HWISE score with the scores for the food item subtracted from it (range 0–33).

† SES score was derived from the first component of a principal component analysis (PCA) conducted with ln(household monthly income+1), ln(livestock wealth+1) and average perceived social status score of household heads (ranging from 1 to 10).

### Household water insecurity and milk intake

HWISE score was not associated with odds of more frequent milk consumption (OR: 1·05, 95 % CI: 0·96, 1·14, *P* = 0·32) (online Supplemental Table 2). In fact, only the PCA-derived SES score was associated with frequency of milk intake (OR: 1·45, 95 % CI: 1·02, 1·95, *P* = 0·035).

## Discussion

The objective of this paper was to understand how HWI relates to HFI among a semi-nomadic pastoralist population living in an arid region of northern Kenya where water availability is extremely limited. We found that the majority of Daasanach households experienced concurrent HWI and HFI, and HWI was strongly related to more frequent experiences with HFI independently of our composite measure of SES. This finding suggests that, although HWI and HFI probably co-occur because of a shared cause (resource scarcity and/or variation in social status), they may also relate to each other independently of financial and social capital.

One pathway through which HWI may exacerbate HFI independently of SES is by affecting which foods can be cooked or prepared. We found that the single HWISE item on the frequency of having to change what was eaten due to water problems was positively associated with HFIAS score independently of other water-related disruptions in daily life, which remained positively associated with HFIAS. Given that the local dish of whole maize and beans requires substantial water to prepare, it may be easier to resort to milled maize porridge when water in the home runs low. However, we lack data on dietary intake to know exactly how water-induced changes in food choice influence food quality or quantity. Future research is needed to better illuminate exactly how different HWI experiences influence HFI directly through impacting food choices and indirectly through its impacts on other aspects of daily life.

We expected that another pathway through which HWI might exacerbate HFI independently of SES would be its influence on access to milk, traditionally a major calorie source for Daasanach. In the semi-permanent communities, even households with substantial wealth in the form of livestock may lack access to their herds as a source of milk because water scarcity and limited vegetation cause many households to send the herds to distant pastures. As a result, the nomadic households travelling with their herds have constant access to milk, but the settled households do not. Contrary to expectations, we found no evidence that recent milk intake was related to HWI. Other circumstances related to livestock ownership, milk production and grazing management at the time of data collection may have added variation that prevented us from detecting a significant trend. Moreover, many traditional pastoralist populations in eastern Africa have transitioned toward diets comprising more cereal grains and legumes, only occasionally supplementing with animal-sourced foods^(34)^. HWI may relate to the number of livestock households keep and have available for selling more than for consumption of milk. Future research investigating the relationship between WI and milk intake should collect data across seasons and include measurements of climate and dietary intake, as well as record which households keep a portion of their herds nearby for milk consumption.

This is one of few known studies to show that HWI relates to HFI independently of other measures of SES. Yet the findings from this study reinforce those from other studies reporting positive multivariable associations between HFIAS and HWISE^(10,11)^ or drinking water source, access and quality^(23)^. Our study findings also corroborate the positive bivariate correlations between FI and other WI scales^(9,12,20)^; water fetching time^(21)^ or as the number of months in the year experiencing water scarcity^(22)^. This trend across studies indicates that the co-occurrence of these two insecurities is common across a wide range of populations with different subsistence patterns and living in distinct environments. However, the magnitude of associations varies across studies, and it is unclear how much of that variation is due to different indicators of WI *v*. different contextual factors, including variation across study sites in climate, season, subsistence patterns, resource access, water infrastructure and other environmental and economic disparities.

Other research reinforcing the idea that WI relates to FI comes from evaluations of FI indicators following water-related intervention studies. For example, one study found that being able to access water on premises following a community water intervention was associated with reporting spending less money and time on water collection than when the water had to be collected off premises from an unimproved source^(18)^. A substantial proportion of households reported spending those financial savings on food (53 %) and time savings on income-generating activities (43 %). This hints at one pathway through which improving water access to households, particularly through reducing time required to fetch water, could reduce HFI. In fact, another study reported a marginal reduction in HFI among communities where a protected faucet was installed relative to households in communities still relying on unprotected spring water^(46)^. While it was unclear why access to an improved water source may have been related to reduced HFI in that study, the authors posited that increased ease of water collection may have contributed.

The findings of the current study have important public health implications as they suggest that both HWI and HFI are serious problems for Daasanach households. The relatively high mean HWISE and HFIAS scores among Daasanach compared with scores reported for other study populations in low- and middle-income countries across the world^(47)^ reinforce previous research suggesting pastoralist populations may be particularly vulnerable to both WI and FI^(28,29,31)^. This could have detrimental health consequences for Daasanach of all ages. FI contributes to stunting and wasting^(48)^, and WI may exacerbate the effects of FI or have independent effects on nutritional status and health^(49–51)^. Furthermore, WI and FI may have lasting harmful syndemic effects on mental health^(8,9,12,50,52)^. Hence, efforts to address HFI need to consider HWI as a major co-insecurity that may exacerbate both HFI and its health consequences.

Failure to consider both insecurities when addressing HFI could also result in suboptimal interventions^(53)^. For example, food aid programmes that distribute staples requiring sufficient water to prepare (e.g. whole grains and legumes) may be less effective in regions with seasonal or chronic water scarcity. More effective interventions may include ones that help restock, diversify, feed and/or water livestock or the development and maintenance of water services that provide reliable support for human, livestock and agricultural needs^(28,29)^. For example, the installation of new community irrigation methods that help support households in efforts to grow food and cash crops, raise livestock and enable food production during dry seasons and drought reduce FI^(54–58)^. Such efforts will be of growing importance given the forecasted impact of increasing droughts on crop production in Africa^(59)^. Pastoralists and other small-scale farming communities will be the most vulnerable to FI and WI in the face of increasing climate variability, given how closely tied their subsistence strategies are to land, vegetation, rainfall and water access.

Further research is needed to address some of the limitations and remaining questions from this study. First, this study does not capture all the present and historical sources of structural, economic and political disadvantage, socio-economic disparities, intertribal conflict and other factors that contribute to HFI and HWI. Second, the cross-sectional nature of our data precludes us from being able to identify causal relationships, confirm directionality, or know if or how HWI, HFI and their relationship may vary across seasons and throughout the year. Moreover, data were collected prior to the COVID-19 pandemic, and it is possible that the co-occurrence of HWI and HFI have since worsened. Third, this study is limited by the overall number of households and communities that were sampled. Given the variation noted across communities even with our sample (online Supplemental Table 1), the findings from this study may not be generalisable to all Daasanach communities or other pastoralist or agro-pastoralist communities. Fourth, the household-level instruments used for assessing HWI and HFI in this population prevent us from capturing variation in experiences that may exist within households between men and women or between adults and children^(52,60–62)^. Likewise, we lack data on the number of females in the household, which would influence how many individuals may have been available to help with water collection and thereby reduce the burden of distant water access. Fifth, the shorter timescale of measuring milk intake (prior week), as well as the limited statistical power due to the small sample size, may have contributed to null relationships with HWI. Finally, the lack of dietary intake data prevents us from gaining a more comprehensive understanding of how HWI may shape food choices, dietary diversity and nutritional intake.

In summary, Daasanach pastoralist households in northern Kenya experience an extremely high prevalence of concurrent moderate-to-severe HWI and HFI. Though financial and social capital are likely a shared cause of both insecurities, the positive relationship between HWI and HFI was independent of our measure of SES that combined income, wealth and perceived social status. Moreover, having to change what was eaten because of water problems were related to HFI independently of other problems with water access and use. Further research is needed to assess if these relationships are causal and to examine the various pathways through which WI may exacerbate FI directly and indirectly. This study highlights the importance of considering WI when developing policies and interventions for addressing FI in settings where limited or unreliable access to sufficient water frequently disrupts daily life, constrains time and restricts what foods can be prepared.

## Supplementary material

For supplementary material accompanying this paper visit https://doi.org/10.1017/S1368980022001689. click here to view supplementary material
