# Supplementary material for: The co-occurrence of water insecurity and food insecurity among Daasanach pastoralists in northern Kenya
Source: Public Health Nutr. 2022 Aug 9;26(3):693–703. doi: 10.1017/S1368980022001689 (PMC9989708; doi:10.1017/S1368980022001689)
Supplement: Supplementary file 1 [file S1368980022001689sup.zip › S1368980022001689sup002.docx]

**ONLINE SUPPLEMENTAL MATERIALS**

The co-occurrence of water insecurity and food insecurity among Daasanach pastoralists in northern Kenya

# Supplemental Text 1. List of questions used for the Household Food Insecurity Access Scale (HFIAS)

The following list of questions used to assess household water insecurity are from the validated Household Food Insecurity Access Scale (HFIAS) questionnaire.^(2)^

1. In the past four weeks, did you worry that your household would not have enough food?
2. In the past four weeks, were you or any household member not able to eat the kinds of foods you preferred because of a lack of resources?
3. In the past four weeks, did you or any household member have to eat a limited variety of foods due to a lack of resources?
4. In the past four weeks, did you or any household member have to eat some foods that you really did not want to eat because of a lack of resources to obtain other types of food?
5. In the past four weeks, did you or any household member have to eat a smaller meal than you felt you needed because there was not enough food?
6. In the past four weeks, did you or any household member have to eat fewer meals in a day because there was not enough food?
7. In the past four weeks, was there ever no food to eat of any kind in your household because of lack of resources to get food?
8. In the past four weeks, did you or any household member go to sleep at night hungry because there was not enough food?
9. In the past four weeks, did you or any household member go a whole day and night without eating anything because there was not enough food?

**Options and scoring for these questions were as follows:**

0... Never (0 times in the last 4 weeks)

1... Rarely (1-2 times in the last 4 weeks)

2... Sometimes (3-10 times in the last 4 weeks)

3... Often or always (11+ times in the last 4 weeks)

Categorization of mild HFI is based on answering “sometimes” or “often or always” to question 1, answering affirmatively at any level to question 2, or answering “rarely” to questions 3 or 4, but answering “never” to questions 5-9.

Categorization of moderate HFI is based on answering “sometimes” or “often or always” to HFIAS questions 3 or 4 or answering “rarely” or “sometimes” to questions 5 or 6, but answering “never” to questions 7-9.

Categorization of severe HFI is based on answering “often or always” to questions 5 or 6 or answering affirmatively (to any degree) to questions 7, 8, or 9.

## Supplemental Text 2. List of questions used for the Household Water Insecurity Experience (HWISE) Scale

The following list of questions used to assess household water insecurity are from the validated Household Water Insecurity Experience (HWISE) Scale questionnaire.^(1)^

1. In the last 4 weeks, how frequently did you or anyone in your household worry you would not have enough water for all of your household needs?
2. In the last 4 weeks, how frequently has your household water supply from your main water source been interrupted or limited (e.g. water pressure, less water than expected)?
3. In the last 4 weeks, how frequently has there not been enough water in the household to wash clothes?
4. **In the last 4 weeks, how frequently have you or anyone in your household had to change what was being eaten because there were problems with water (e.g. for washing foods, cooking, etc.)?**
5. In the last 4 weeks, how frequently have you or anyone in your household had to go without washing hands after dirty activities (e.g., defecating or changing diapers, cleaning animal dung) because of problems with water?
6. In the last 4 weeks, how frequently have you or anyone in your household had to go without bathing because of problems with water (e.g., lack of water, unclean water, etc.)?
7. In the last 4 weeks, how frequently has your or anyone in your household’s day been interrupted by problems with your water situation, such as problems getting or distributing water within the household? (Activities that may have been interrupted include caring for others, doing household chores, or attending social events, etc.)
8. In the last 4 weeks, how frequently did you or anyone in your household feel angry about your water situation?
9. In the last 4 weeks, how frequently has there not been as much water to drink as you would like for you or anyone in your household?
10. In the last 4 weeks, how frequently have you or anyone in your household gone to sleep thirsty because there wasn’t any water to drink?
11. In the last 4 weeks, how frequently has there been no useable water whatsoever in your household?
12. In the last 4 weeks, how frequently have problems with water caused you or anyone in your household to feel ashamed/excluded/stigmatized?

Options and scoring for these questions were as follows:

0... Never (0 times in the last 4 weeks)

1... Rarely (1-2 times in the last 4 weeks)

2... Sometimes (3-10 times in the last 4 weeks)

3... Often or always (11+ times in the last 4 weeks)

## Supplemental Table 1. Household characteristics by community numbered by closest to farthest from the nearest commercial town (n=136 households)

|  | **Community Number** | | | | | | | | | | | | |
| --- | --- | --- | --- | --- | --- | --- | --- | --- | --- | --- | --- | --- | --- |
|  | 1 |  | 2 |  | 3 |  | 4 |  | 5 |  | 6 |  | 7^a^ |
|  |  |  |  |  |  |  |  |  |  |  |  |  |  |
| Number of households | 14 |  | 24 |  | 23 |  | 13 |  | 28 |  | 22 |  | 12 |
|  |  |  |  |  |  |  |  |  |  |  |  |  |  |
| **Sociodemographic characteristics** |  |  |  |  |  |  |  |  |  |  |  |  |  |
| Type of housing | Semi-permanent |  | Semi-permanent |  | Semi-permanent |  | Semi-permanent |  | Semi-permanent |  | Semi-permanent |  | Temporary |
| Number of times moved in the previous year | 0.6 (0.9) |  | 1.6 (3.0) |  | 2.3 (2.8) |  | 3.5 (3.5) |  | 5.6 (8.6) |  | 5.0 (5.3) |  | 12.2 (7.6) |
| Female household head age in years, mean (SD) | 47.0 (19.1) |  | 36.6 (12.6) |  | 35.5 (12.7) |  | 34.2 (6.5) |  | 31.1 (9.7) |  | 32.1 (10.2) |  | 29.8 (9.7) |
| Number of children <16 years in household, mean (SD) | 3.1 (2.7) |  | 3.5 (2.3) |  | 5.3 (2.9) |  | 5.8 (1.3) |  | 4.5 (2.2) |  | 4.5 (2.5) |  | 4.7 (2.5) |
| Previous month's household income, USD, median (IQR) | 15.5 (11.0, 21.0) |  | 14.5 (11.0, 18.0) |  | 18.0 (15.0, 21.0) |  | 17.0 (17.0, 18.0) |  | 18.0 (16.5, 19.0) |  | 21.5 (18.0, 23.0) |  | 17.0 (15.0, 17.5) |
| Household monetary value of livestock, USD, median (IQR) | 19.0 (17.0, 25.0) |  | 14.5 (11.5, 21.5) |  | 17.0 (15.0, 24.0) |  | 21.0 (18.0, 24.0) |  | 18.5 (15.0, 24.0) |  | 23.0 (22.0, 25.0) |  | 24.5 (21.5, 27.0) |
| Perceived social status  (range: 1-10/worst-best) | 2.8 (1.6) |  | 3.1 (1.8) |  | 3.2 (2.3) |  | 2.5 (1.5) |  | 3.1 (1.8) |  | 2.9 (2.1) |  | 6.6 (2.1) |
|  |  |  |  |  |  |  |  |  |  |  |  |  |  |
| **Indicators of food insecurity** |  |  |  |  |  |  |  |  |  |  |  |  |  |
| HFIAS score, mean (SD) | 16.1 (6.2) |  | 14.0 (7.3) |  | 18.0 (4.9) |  | 18.3 (3.4) |  | 17.6 (3.5) |  | 20.8 (3.2) |  | 16.2 (3.3) |
| Moderate food insecurity  (% of households) ^b^ | 7.1% |  | 4.2% |  | 0.0% |  | 0.0% |  | 0.0% |  | 0.0% |  | 8.3% |
| Severe food insecurity  (% of households) ^b^ | 92.9% |  | 87.5% |  | 100.0% |  | 100.0% |  | 100.0% |  | 100.0% |  | 91.7% |
|  |  |  |  |  |  |  |  |  |  |  |  |  |  |
| Frequency of milk intake in the previous week |  |  |  |  |  |  |  |  |  |  |  |  |  |
| None | 21.4% |  | 16.7% |  | 78.3% |  | 61.5% |  | 46.4% |  | 27.3% |  | 0.0% |
| Less than daily | 71.4% |  | 45.8% |  | 0.0% |  | 15.4% |  | 17.9% |  | 13.6% |  | 0.0% |
| Daily | 7.1% |  | 37.5% |  | 21.7% |  | 23.1% |  | 35.7% |  | 59.1% |  | 100.0% |
|  |  |  |  |  |  |  |  |  |  |  |  |  |  |
| **Indicators of water insecurity** |  |  |  |  |  |  |  |  |  |  |  |  |  |
| HWISE score, mean (SD) | 20.3 (5.9) |  | 15.3 (8.4) |  | 19.9 (7.1) |  | 21.0 (4.4) |  | 19.9 (6.3) |  | 23.1 (5.5) |  | 24.8 (3.8) |
| HWISE score ≥12 and <24  (% of households) | 64.3% |  | 54.2% |  | 69.6% |  | 69.2% |  | 64.3% |  | 50.0% |  | 41.7% |
| HWISE score ≥24  (% of households) | 28.6% |  | 20.8% |  | 30.4% |  | 30.8% |  | 32.1% |  | 45.5% |  | 58.3% |
|  |  |  |  |  |  |  |  |  |  |  |  |  |  |
| Percent of households having to change what was eaten because of water problems in the previous four weeks |  |  |  |  |  |  |  |  |  |  |  |  |  |
| Never/rarely (0-2 times) | 50.0% |  | 66.7% |  | 21.7% |  | 7.7% |  | 46.4% |  | 18.2% |  | 16.7% |
| Sometimes/often/always (3+ times) | 50.0% |  | 33.3% |  | 78.3% |  | 92.3% |  | 53.6% |  | 81.8% |  | 83.3% |
|  |  |  |  |  |  |  |  |  |  |  |  |  |  |
| Hours/day spent fetching water | 4.1 (2.1) |  | 4.7 (2.8) |  | 5.2 (2.4) |  | 3.2 (1.8) |  | 3.4 (1.7) |  | 4.2 (2.4) |  | 5.0 (2.0) |
| Distance from nearest water source (meters) | 357.8 (59.0) |  | 392.4 (90.5) |  | 739.6 (139.6) |  | 426.5 (121.2) |  | 888.8 (385.6) |  | 378.3 (50.0) |  | 2765.6 (0.0) |
|  |  |  |  |  |  |  |  |  |  |  |  |  |  |

^a^ Community 7 was the one fully nomadic community. They moved very few weeks. At the time of data collection, every household relied on daily milk intake as a major source of sustenance, and they all collected water from the same location.

^b^ See Supplemental Text 1 for HFIAS scoring and categorization of moderate and severe food insecurity

**Supplemental Table 2. Multivariable ordered logistic regression model of the odds of more frequent milk intake among adults in relation to HWISE score among the six settled communities (n=124 households) ^a^**

|  |  |  |  |  |
| --- | --- | --- | --- | --- |
|  | OR | (95% CI) | *P* | *Pseudo R^2^* |
|  |  |  |  |  |
| HWISE score | 1.05 | (0.96, 1.14) | 0.28 |  |
|  |  |  |  |  |
| PCA-derived SES score ^b^ | 1.52 | (1.12, 2.05) | 0.0074 |  |
|  |  |  |  |  |
| Age of female household head (years) | 0.99 | (0.96, 1.03) | 0.74 |  |
| Number of children in household | 0.92 | (0.71, 1.20) | 0.55 |  |
| Times moved in the previous years | 1.02 | (0.97, 1.07) | 0.54 |  |
| Hours/day spent collecting water | 1.03 | (0.86, 1.22) | 0.75 |  |
|  |  |  |  |  |
|  |  |  |  | 0.114 |

HWISE, Household Water Insecurity Experience

Results from a multivariable ordered logistic regression model with robust SEs regressing adult milk intake (categorized as none, 1-6 days/week, and ≥7 days/week in the previous week) on HWISE score, adjusting for covariates listed above and community fixed effects while also clustering on community.

^a^ Excluding the one nomadic community (n=12 households) in which there was no variation in milk intake.

^b^ SES score based on the first component of a principal component analysis conducted with ln(household monthly income+1), ln(livestock wealth+1), and average perceived social status score of household heads (ranging from 1-10).

**Supplemental References**

1. Young SL, Boateng GO, Jamaluddine Z, et al. (2019) The Household Water InSecurity Experiences (HWISE) Scale: development and validation of a household water insecurity measure for low-income and middle-income countries. *BMJ Global Health* **4**, e001750.

2. Coates J, Swindale A & Bilinsky P (2007) *Household Food Insecurity Access Scale (HFIAS) for Measurement of Household Food Access: Indicator Guide (v. 3)*. Washington, D.C.: Food and Nutrition Technical Assistance Project, Academy for Educational Development.
